# Obituary

**Published:** 1885-02

**Authors:** 


### OBITUARY.

### E. H. GIBBS, A. M., M. D.

Died January 5th, 1885.

Dr. Gibbs was born at Ovid, on Seneca Lake, N. Y., in the year 1823.

He was a graduate of Union College, although his first collegiate year was spent at Geneva College. He studied medicine at the Buffalo Medical College, under Drs. Frank Hamilton and Austin Flint.

Upon taking his degree, he went to France, where he studied for three years under the greatest physicians and surgeons of the time.

Returning to America he practiced medicine successfully for many years. In 1871 he became the partner of Dr. W. W. Hall, the founder of the JOURNAL OF HEALTH, and after Dr. Hall's death, in 1876, took entire charge of the JOURNAL, which he conducted with excellent judgment until the beginning of the present year.

